# The diversity of peritoneal dialysis care trajectories: A study based on the REIN registry and SNDS database

**DOI:** 10.1371/journal.pone.0326745

**Published:** 2025-08-05

**Authors:** Bruno Legendre, Thierry Lobbedez, Cécile Couchoud, Antoine Lanot, Anne Kolko, Cécile Courivaud, Annabel Boyer, Clémence Béchade

**Affiliations:** 1 Normandie Univ, UNICAEN, CHU de Caen Normandie, Néphrologie, Caen, France; 2 INSERM U1086, “ANTICIPE”, Caen, France; 3 AUB Santé Dialyse, Avranches, France; 4 Agence de la biomédecine, REIN registry, La Plaine Saint-Denis, Ile de France, France; 5 AURA Paris, Paris, France; 6 Néphrologie, Centre Hospitalier Universitaire de Besançon, Besançon, France; 7 Université de Franche-Comté, CHU Besançon, EFS, INSERM, UMR RIGHT, Besançon, France; Phramongkutklao College of Medicine, THAILAND

## Abstract

**Introduction:**

The integrated care model considers sequences of kidney replacement therapies rather than individual modalities. Data from conventional registries describing trajectories are partial. The aim was to provide a complete description of the peritoneal dialysis pathway.

**Methods:**

Patients undergoing peritoneal dialysis in France between January 1, 2009, and December 31, 2019, were included. Data from the national REIN (Renal Epidemiology and Information Network) registry and the SNDS (Administrative Database of Outpatient and Inpatient Care Consumption) database were used. Patient trajectories and status (peritoneal dialysis, hemodialysis, mixed and hybrid dialysis, kidney transplantation, and death) were reconstructed. Each dialysis session, even a single day, was recorded. The trajectory was described using a Sankey diagram.

**Results:**

A total of 5,053 patients in the REIN registry and the SNDS database underwent peritoneal dialysis at some point. The Sankey diagram showed the great diversity and complexity of care pathways. Only 1,652 (33%) of patients underwent peritoneal dialysis only. A total of 1,807 (36%) patients changed kidney replacement therapy 2 times or more. There was high permeability between peritoneal dialysis and hemodialysis: 1,358 (27%) patients transferred from hemodialysis to peritoneal dialysis and 2,018 (40%) transferred from peritoneal dialysis to hemodialysis. A total of 251 (5%) patients underwent hybrid dialysis. A total of 498 (10%) patients experienced an unstable period of mixed dialysis and managed to return to peritoneal dialysis for a significant time (median of 339 days). The causes of transfers were not available.

**Conclusion:**

Our results describe a more precise view of the trajectories of peritoneal dialysis patients compared to data from conventional registries. Peritoneal dialysis is a component of a multimodal pathway, as two thirds are likely to receive another kidney replacement therapies. Ongoing information about other kidney replacement therapies regimens seems necessary for peritoneal dialysis patients. The organization of each dialysis center must integrate and facilitate these transfers.

## Introduction

Patients with end-stage kidney disease (ESKD) must choose between different kidney replacement therapies (KRTs). The integrated care model considers trajectories and sequences of therapies rather than individual modalities. However, most patients will experience several modalities [1–5] over the course of their ESKD. PD patients are very likely to change KRT modality. In France, 38% of patients have left the technique at one year [6]. This result is consistent with figures found in many other countries, where fewer than 50% of patients remained on PD 2–3 years after PD initiation [5].

It is admitted that PD and hemodialysis (HD) have comparable results. These 2 techniques are complementary and can be used consecutively over time [7]. However, the transition phases are at risk of significant morbidity and mortality [8–10] if they are not planned in advance. Even if the data on this point are scarce, this postulate is accepted by the nephrology community [5].

Data describing and understanding the different care trajectories are lacking [1,5]. Most of these studies are based on data from registries. However, the definition of transfer from one technique to another is open to debate and not all registries have the same definition of transition [11]. Some transitions are also underreported in classic national registries, as short transitions (< 2 months in the REIN registry) are often not subject to mandatory reporting. The INTEGRATED group defines transition as a switch to another KRT for at least 30 days. Sensitivity analyses with 60, 90 and 180 days have been proposed [5,12].

These definitions could seem far from the patient’s experience, for whom the transfer from PD to HD remains a major event, even for 1 day [13]. However, it is almost impossible to identify every event in registries based on a retrospective collection of clinical data, and a prospective study aiming to follow day by day a cohort of PD patients would be difficult to perform.

Our hypothesis is that the trajectories of patients who undergo peritoneal dialysis are particularly diverse and complex. Given that the data from registries would not be precise enough to describe in detail every rotation between HD and PD, we used another source of information to capture all HD sessions performed in a population of PD patients. Access to increasingly exhaustive databases allows us to reconstruct patient care trajectories as closely as possible [14]. We performed a linkage between an exhaustive national database, the French National Health Data System, database that contains all dialysis service reimbursements, and the REIN registry, to describe in detail the trajectories of patients treated by PD in France.

The main objective of this study was to extensively describe the different trajectories of patients treated at least one time with PD in France to determine the complexity of patient pathways across dialysis modalities and eventually to confirm that PD and HD are complementary methods for integrative care.

## Materials and methods

### Statement of ethics

The REIN registry was approved by the relevant French committees, the Comité consultatif sur le traitement de l’information en matière de recherche (CCTIRS) and the Commission nationale de l’informatique et des libertés (CNIL N° 903188). Patients are informed about the registration in the REIN registry and their right to not participate (opt out) by the nephrology clinic.

Additionally, the permission to access the raw data used in our study (the SNDS data linked with the REIN registry data) was granted by the French Data Protection Authority (Commission Nationale Informatique et Liberté, CNIL authorization N° 917021). All data used in this study was anonymized before its use. All methods in this study were performed in accordance with relevant guidelines and regulations.

### Study population and databases

All patients aged older than 18 years who started maintenance dialysis in France between 01/01/2009 and 31/12/2019 in the Renal Epidemiology and Information Network (REIN) registry were identified and matched to the SNDS database. All patients who underwent PD at any time in the SNDS database were included in this study. The REIN registry collects data on all patients with ESKD in France when they start their first KRT. It relies on a network of nephrologists, epidemiologists, patients and public health representatives coordinated regionally and nationally. Disease-specific clinical data were obtained [15,16]. To obtain information on health consumption, these patients were matched to the SNDS (Système National des Données de Santé [SNDS]) database. The SNDS is a medico-administrative database used for the reimbursement of all non-hospital-based outpatient healthcare and all hospital activity. Reporting is done at different levels, sometimes automatically, but is mandatory for healthcare operators to be reimbursed. As regards reimbursement for KRTs, the declaration is made by the healthcare structures. The SNDS covers 99% of the French population [17]. Each dialysis session or care consumption is recorded. The strategy used to do this record linkage between REIN and SNDS is a deterministic procedure, based on a set of matching rules for selected identifiers; it has been previously published [18]. A record pair will only be considered to match if the two records agree on all identifiers of the rules. The algorithm includes 24 matching rules, or steps, with progressively less strict conditions. This iterative deterministic approach is similar to the one used in other international linkages, such as the Clinical Practice Research Datalink (CPRD) in the UK, and the US Surveillance, Epidemiology and End Results (SEER)-Medicare.

Patients who underwent preemptive transplantation were excluded. Patients who were not matched between the REIN registry and the SNDS database were excluded.

Each patient included in this study was followed up until death or the end of the observation period (31/12/2020). The data were extracted from the REIN registry on 28th June 2022. The authors did not have access to information that would allow individual participants to be identified.

### Dialysis modalities

As the SNDS database is exhaustive, each HD and PD session was reported, and information on dialysis treatment was provided day-by-day. Outpatient and inpatient dialysis sessions, in public or private structure, were available.

A patient was considered to be on hybrid dialysis when he switched between HD and PD, each week for at least 3 months. This reflects a period of stability in the patient’s trajectory. This concept is used, particularly in Japan, to overcome insufficient small solute clearance or fluid overload problems [19]. The common practice is PD prescribed for 4–5 days along with HD prescribed once or twice per week [20,21].

We introduced the new notion of mixed dialysis for patients who alternate between HD and PD, recurrently, over a short period of time, due to clinical complications. For mixed dialysis, several thresholds were tested for the number of switches between HD and DP. The number of 2 changes, i.e., 3 dialysis modalities, was retained after reading the trajectories of the most complex patients. The definition used was a succession of 3 different modalities, where the patient remained in each modality for less than a month.

These two modalities involved few patients, but their definitions allowed us to make trajectories more readable (S1 Fig).

We calculated the number of consecutive days spent on each KRT modality for each patient, until the next modality, death or end of follow-up. We counted the number of KRTs per patient, even for 1 day spent in a KRT.

### Events of interest

The main objective was to describe transitions between states among patients treated for at least one day with PD. A transition was defined as a sequence between states. The following six states were considered: HD, PD, hybrid dialysis, mixed dialysis, transplantation or death. A patient who had an HD then PD then HD trajectory was considered to have had 3 states. Therefore, every transition in the care trajectory was analyzed.

### Covariates

The REIN registry records demographic data (sex, age, body mass index (BMI), activity (nonworking people, including pensioners, homemakers and unemployed people)) at KRT initiation.

It records clinical characteristics at KRT start (disability, walking, underlying nephropathy, diabetes mellitus, cardiovascular disease (defined as the following conditions: stroke or transient ischemic attack or dysrhythmia or peripheral vascular disease or coronary heart disease), congestive heart failure, chronic respiratory disease, cirrhosis, and active cancer) and dialysis characteristics (including emergency initiation [defined as the first dialysis session performed immediately (<24h after assessment by a nephrologist), due to life-threatening conditions: hyperhydration, hyperkalemia, acidosis, confusion, pericarditis, anemia], start on temporary HD catheter, hemoglobinemia and albuminemia before dialysis initiation).

The SNDS database included the following variables: each PD and HD sessions performed at home or in facility based and hospitalizations for cardiovascular disease (from 6 months before to 6 months after the first transition).

### Statistical analysis

Patient characteristics at the initiation of dialysis were described by percentages for categorical variables and medians with interquartile ranges for continuous variables. The distribution of the variables did not follow a normal distribution. Missing data were considered as such. The PD to DEATH, PD to HD, HD to PD and PD to KT groups were compared using the Kruskal-Wallis rank sum test and Pearson’s Chi-squared test.

### Patient trajectories

**Fig 1:**
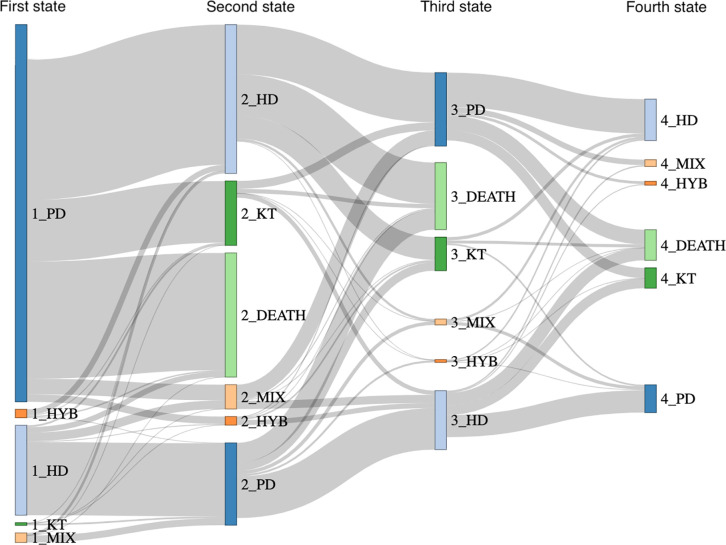
Sankey diagram depicting the flow of consecutive KRTs used by the 5,053 PD patients (limited to the first 4 KRTs). The size of each node and arc is proportional to the number of patients. The first column of nodes represents the distribution of the first state (PD, HD, mixed and hybrid dialysis), the second column represents the distribution of the second state (PD, HD, mixed and hybrid dialysis, kidney transplantation, death), *etc*. Time is not represented in this diagram. KRT: kidney replacement therapy. PD: peritoneal dialysis. HD: hemodialysis. KT: kidney transplantation. HYB: hybrid dialysis. MIX: mixed dialysis.

The completeness of the SNDS database can lead to very complex patient trajectories. A Sankey flow chart was used to show the flow and changes in dialysis techniques (Fig 1). To visualize the trajectories and changes over time, the nodes were organized vertically and by step, to create an alluvial diagram. The transition from one of these modalities to the other is easily represented on a Sankey diagram [22]. The size of each node and arc is proportional to the number of patients. The first column of nodes represents the distribution of the cohort among the first state, the second column represents the distribution of the second state, etc. The proportion of a node that is not followed by an arc means that this proportion of patients is still receiving the technique at the end of the observation period.

The nodes corresponded to the following states: HD, PD, kidney transplantation, death, hybrid dialysis and mixed dialysis. To avoid making the diagram too complex, we limited the number of steps to the first 4 states. Such a diagram does not provide information on the time spent on the different modalities.

To provide this information, we also reported the median time spent on each dialysis modality. We decided to detail the 4 most frequent first transitions and the characteristics of patients who underwent mixed and hybrid dialysis.

Analyses were performed with R software, version 2023.03.0 + 386 [23]. The network3D package was used to construct the Sankey diagram.

## Results

### Participants

**Fig 2:**
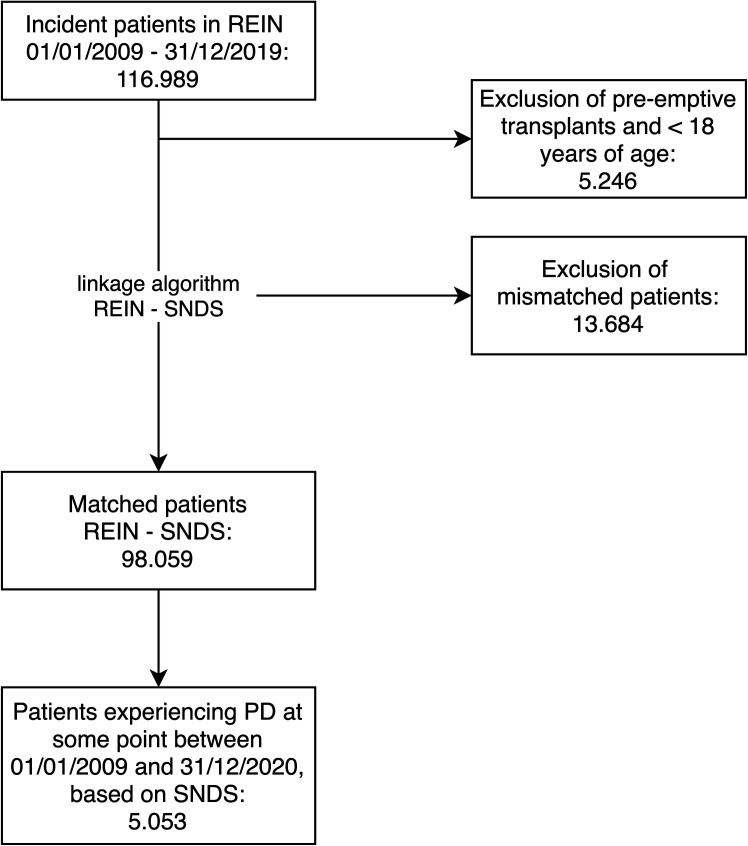
Flowchart of the selection process for patients who underwent PD using the REIN – SNDS linkage algorithm. PD: peritoneal dialysis.

The selection of patients who were treated with PD for at least one day is shown in Fig 2. A total of 13,684 (12%) of the 116,989 incidental patients in the REIN registry were not matching in the SNDS database. Among the 98,059 patients who started dialysis and were identified in both the REIN and SNDS databases, 5,053 (5%) underwent PD at some point. This number is similar to the prevalence of PD in the REIN 2021 report: 6% [24]. Overall, the patients who underwent PD had a median age of 70 years, 61% were male, 37% were diabetic and 47% had a cardiovascular disease (Table 1).

**Table 1:** Characteristics of patients according to their first transition.

	Overall	PD to DEATH	PD to HD	HD to PD	PD to KT	p-value^2^
	n = 5,053	n = 1,215	n = 1,452	n = 757	n = 638	
**Clinical characteristics at KRT start** ^**1**^						
Age (years)	70 (56, 81)	81 (74, 85)	69 (56, 78)	69 (53, 80)	51 (38, 63)	<0.001
Sex (M)	3,071 (61%)	744 (61%)	891 (61%)	470 (62%)	362 (57%)	0.2
Professional activity						<0.001
Working	705 (14%)	15 (1%)	190 (13%)	123 (16%)	232 (36%)	
Non-working	3,200 (63%)	962 (79%)	922 (63%)	472 (62%)	210 (33%)	
Missing	1,148 (23%)	238 (20%)	340 (23%)	162 (21%)	196 (31%)	
Underlying nephropathy						<0.001
Vascular or hypertensive nephropathy	1,294 (26%)	423 (35%)	369 (25%)	192 (25%)	79 (12%)	
Diabetic nephropathy	983 (19%)	274 (23%)	318 (22%)	144 (19%)	50 (8%)	
Glomerulonephritis	697 (14%)	66 (5%)	206 (14%)	97 (13%)	171 (27%)	
Polycystic kidneys	313 (6%)	21 (2%)	88 (6%)	34 (5%)	104 (16%)	
Other	834 (17%)	137 (11%)	224 (15%)	139 (18%)	162 (25%)	
Unknown	932 (18%)	294 (24%)	247 (17%)	151 (20%)	72 (11%)	
Diabetes	1,866 (37%)	575 (47%)	560 (39%)	273 (36%)	85 (13%)	<0.001
Missing	73 (1%)	17 (1%)	12 (1%)	8 (1%)	15 (2%)	
Cardiovascular disease	2,359 (47%)	858 (71%)	633 (44%)	351 (46%)	89 (14%)	<0.001
Missing	147 (3%)	34 (3%)	40 (3%)	24 (3%)	22 (3%)	
Congestive heart failure	1,386 (27%)	568 (47%)	356 (25%)	228 (30%)	31 (5%)	<0.001
Missing	136 (3%)	40 (3%)	40 (3%)	21 (3%)	16 (3%)	
BMI (kg/m2)	25.4 (22.4, 28.8)	25.6 (22.6, 29.2)	25.8 (22.7, 29.3)	24.6 (22.1, 28.1)	24.2 (21.2, 27.5)	<0.001
Missing	1,019 (20%)	287 (24%)	292 (20%)	156 (21%)	100 (16%)	
Smoking	1,665 (33%)	357 (29%)	517 (36%)	279 (37%)	181 (28%)	<0.001
Missing	869 (17%)	289 (24%)	234 (16%)	122 (16%)	94 (15%)	
Disability	570 (11%)	195 (16%)	134 (9%)	94 (12%)	31 (5%)	<0.001
Missing	329 (7%)	109 (9%)	92 (6%)	41 (5%)	34 (5%)	
Walking						<0.001
Total disability	141 (3%)	71 (6%)	20 (1%)	28 (4%)	1 (0%)	
Need for another person	455 (9%)	199 (16%)	113 (8%)	77 (10%)	4 (1%)	
Autonomous walking	3,797 (75%)	777 (64%)	1,134 (78%)	565 (75%)	533 (84%)	
Missing	660 (13%)	168 (14%)	185 (13%)	87 (11%)	100 (16%)	
**Dialysis characteristics at KRT start** ^**1**^						
Emergency start of dialysis	734 (15%)*	88 (7%)*	104 (7%)*	341 (45%)	38 (6%)*	<0.001
Missing	507 (10%)*	162 (13%)*	135 (9%)*	41 (5%)	103 (16%)*	
HD start on catheter	NC	NC	NC	569 (75%)	NC	<0.001
Missing	NC	NC	NC	80 (11%)	NC	
Albuminemia (g/L)	34.9 (30.0, 38.4)	34.0 (30.0, 37.0)	35.0 (31.0, 38.9)	32.0 (27.8, 36.3)	37.0 (33.3, 41.0)	<0.001
Missing	1,659 (33%)	381 (31%)	482 (33%)	248 (33%)	234 (37%)	
Hemoglobinemia < 10 g/dL	1,429 (28%)	315 (26%)	364 (25%)	343 (45%)	107 (17%)	<0.001
Missing	730 (14%)	156 (13%)	203 (14%)	114 (15%)	126 (20%)	
**Hospitalizations**						
Hospitalization for CV disease (6 months before and 6 months after transition)	883 (17%)	277 (23%)	307 (21%)	166 (22%)	26 (4.1%)	<0.001

1 Median (IQR); n (%).

² Kruskal-Wallis rank sum test; Pearson’s Chi-squared test. p-value comparing PD to DEATH, PD to HD, HD to PD and PD to KT.

* in France, very few emergency start-ups are carried out with PD.

KRT: kidney replacement therapy; PD: peritoneal dialysis; HD: hemodialysis; KT: kidney transplantation; BMI: body mass index; NC: not concerned; CV: cardio vascular.

Among those 5,053 PD patients, 2,903 (57%) underwent HD at some point (including mixed and hybrid dialysis) and 1,393 (28%) underwent transplantation. A total of 1,652 (33%) patients received only PD without any other KRT during the observation period (still in PD at the end of the observation period [365] or died while on PD [1,287]).

The time trend in patient numbers was as follows: At start-up, of the 5,053 patients, 5,026 were on dialysis, including 3,898 on PD. At 1 year, among the 4,415 living patients, 4,115 were on dialysis, including 3,305 on PD. At 3 years, among the 3,359 living patients, 2,440 were on dialysis, including 1,456 on PD.

**Fig 3:**
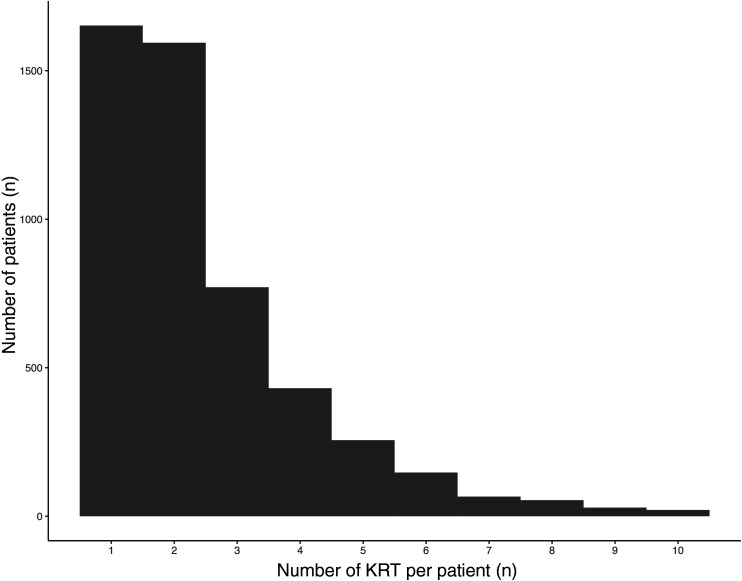
Number of KRTs (PD, HD, mixed and hybrid dialysis, KT) per patient, limited to 10. KRT: kidney replacement therapy. PD: peritoneal dialysis. HD: hemodialysis. KT: kidney transplantation.

These PD patients underwent a median of 2 KRTs (IQR 1–3) over the study period (Fig 3). A total of 1,807 patients (36%) underwent 3 KRTs or more.

### The complexity and diversity of PD trajectories

The flow of technique changes is shown in the Sankey diagram in Fig 1. A change in technique even for a single dialysis session is represented. Time is not represented in this diagram, so short or long changes in the technique have the same representation.

There were 1,880 transfers from HD to PD (for 1,358 (27%) patients) and 2,683 transfers from PD to HD (for 2,018 (40%) patients). The Sankey diagram highlights the complexity and diversity of peritoneal dialysis patient trajectories. In particular, the figure shows the permeability and back-and-forth relationships between PD and HD.

### The first 4 major transitions

For the first transition, the Sankey diagram revealed 4 main trajectories: PD to HD (n = 1,452), PD to death (n = 1,215), HD to PD (n = 757) and PD to transplantation (n = 638). Patient characteristics are presented in Table 1.

Patients who switched from PD to HD had a median PD duration of 394 days. Median age was 69 and 39% had diabetes mellitus. When looking at the rest of their career, the third modality after PD-HD was mainly PD (35%). The characteristics of PD-HD-PD patients are presented in S1 Table. The median time spent in HD in that sub-group was 9 days and median time with the third modality (PD) was 173 days.

A total of 757 patients were transferred from HD to PD, and the median time spent on HD was 53 days. Patients who underwent HD to PD transfer as a first transition were prone to unplanned dialysis initiation (high emergency (45%) and catheter start-up (75%), lower albuminemia (median at 35g/L), lower hemoglobinemia (45% had hemoglobinemia < 10 g/dL)), than other patients were.

More than 20% of patients were hospitalized for cardiovascular disease between 6 months before and 6 months after their first transition.

### Mixed and hybrid dialysis

The characteristics of the patients who underwent mixed or hybrid dialysis at some time are shown in Table 2. From 2009 to 2019, 251 (5%) patients underwent hybrid dialysis in France. Among the 251, 87 (35%) started with this modality. Hybrid dialysis appeared to be more common in diabetic patients (42%), with a lower frequency of cardiovascular disease (39%). As shown in Table 3, the median time spent on hybrid therapy after initiation of PD (line “PD-HYB” in Table 3) was 386 days.

**Table 2:** Characteristics of patients experimenting at least once either mixed or hybrid dialysis.

	Mixed dialysis, n = 498^1^	Hybrid dialysis, n = 251^1^
**Clinical characteristics at KRT start**		
Age (years)	66 (50, 76)	69 (56, 77)
Sex (M)	323 (65%)	153 (61%)
Professional activity		
Working	92 (18%)	33 (13%)
Non-working	303 (61%)	147 (59%)
Missing	103 (21%)	71 (28%)
Underlying nephropathy		
Vascular or hypertensive nephropathy	104 (21%)	51 (20%)
Diabetic nephropathy	102 (20%)	59 (24%)
Glomerulonephritis	95 (19%)	38 (15%)
Polycystic kidneys	37 (7.4%)	22 (8.8%)
Other	72 (14%)	49 (20%)
Unknown	88 (18%)	32 (13%)
Diabetes	174 (35%)	106 (42%)
Missing	5 (1.0%)	3 (1.2%)
Cardiovascular disease	208 (42%)	99 (39%)
Missing	13 (2.6%)	3 (1.2%)
Congestive heart failure	111 (22%)	41 (16%)
Missing	8 (1.6%)	2 (0.8%)
BMI (kg/m2)	25.6 (22.5, 29.5)	25.6 (23.0, 30.1)
Missing	89 (18%)	30 (12%)
Smoking	176 (35%)	90 (36%)
Missing	45 (9.0%)	18 (7.2%)
Disability	65 (13%)	40 (16%)
Missing	16 (3.2%)	11 (4.4%)
Walking		
Total disability	11 (2.2%)	4 (1.6%)
Need for another person	29 (5.8%)	11 (4.4%)
Autonomous walking	412 (83%)	216 (86%)
Missing	46 (9.2%)	20 (8.0%)
**Dialysis characteristics at KRT start**		
Albuminemia (g/L)	35 (29, 38)	36 (32, 40)
Missing	162 (33%)	48 (19%)
Hemoglobinemia < 10 g/dL	173 (35%)	71 (28%)
Missing	64 (13%)	14 (6%)

^1^Median (IQR); n (%).

KRT: kidney replacement therapy; PD: peritoneal dialysis; HD: hemodialysis; KT: kidney transplantation; BMI: body mass index; NC: not concerned.

**Table 3:** Number of patients and median time spent in each modality according to kidney replacement therapy trajectories.

KRT trajectories	Number of patients	Median time spent in the 1^st ^modality (days)	Median time spent in the 2^nd ^modality (days)
PD – DEATH	1,215	588	*NC*
PD – KT	638	522	1,340
PD – HD – PD	506	262	9
PD – HD – DEATH	412	427	553
PD – HD – KT	228	414	469
PD – MIX – PD	87	450	30
PD – MIX – HD	60	555	27
PD – HYB	77	452	386
HD – PD – HD	382	56	122
HD – PD – DEATH	186	39	520
HD – MIX – PD	64	74	24
HD – MIX – HD	23	61	29
MIX – PD	72	36	339
MIX – HD	25	50	212
HYB – HD	59	453	60

PD: peritoneal dialysis; HD: hemodialysis; KT: kidney transplantation; MIX: mixed dialysis; HYB: hybrid dialysis; NC: not concerned; KRT: kidney replacement therapy.

Mixed dialysis was a short period of transition that then resulted in a more stable period. The median time spent on mixed dialysis was 33 days (IQR 21–48). A total of 498 (10%) patients underwent mixed dialysis in France. Even in this type of trajectory, patients were then treated with PD for a considerable period of time. The median time on PD was 339 days for the subgroup of patients treated with mixed dialysis and then PD (Table 3, line “MIX-PD”). The median time on PD was 433 days in the subgroup of patients treated with HD, mixed dialysis, and then PD.

## Discussion

To the best of our knowledge, this is the first extensive description of the trajectory of PD patients based on an exhaustive national database in which every dialysis session was recorded. Our study showed that PD is integrated into a complex KRT pathway. Indeed, only 33% of patients underwent PD as the only KRT. Patients treated for at least one day with PD had a median of 2 KRTs during their care trajectory. The patient profile according to the first transition varied significantly, suggesting different trajectories according to comorbidities. It confirms that individuals with ESKD should be informed about every dialysis modality, as they are likely to undergo PD and HD [7]. The SONG-PD report has shown that one of the main concern of patients undergoing PD is *“how long they can stay on PD”* and how to *“avoid transfer to hemodialysis, which were critical for consideration in identifying the core outcome domains”* [13,25]. At the center level, dialysis centers need to anticipate the fact that the majority of PD patients will experiment with another KRT (in particular HD). There should be an early reflection process to organize the pathway in each center, especially for hemodialysis referrals, which are very frequent. Our study highlights that the transfer from HD to PD may be underestimated in registries, as short transitions are often not subject to reporting. We demonstrated that HD to PD transfer was not a rare event, as it occurred in 1,358 (27%) patients during the study period. Other studies found similar figure: Nessim et al. [26] found that 9,404 (71%) patients started directly in PD and 3,757 (29%) after a period in HD. On the other hand, in the United States Renal Data System (USRDS), switches from HD to PD occured in only 3.5% to 4.7% of patients without early PD experience [27]. Patients who switched in our study from HD to PD as a first transition were more likely to have unplanned dialysis initiation, as 45% started as an emergency situation and 75% with an HD catheter). For comparison, in the latest REIN report, 29% of all incident dialysis patients started in emergency and 58% started with an HD catheter [24] This is a matter of concern since unplanned dialysis initiation is associated with additional morbidity [8,9] and the time spent on HD has an impact on the outcome of PD patients [28–30]. One may argue that transition care units could improve the management of unplanned dialysis starters [31–36].

The switch from PD to HD was the first transition, experienced by 1,452 of the 3,898 (37%) patients starting PD first in our study. These patients had a median PD duration of 394 days. In a study from the Australia and New Zealand Dialysis and Transplant Registry (ANZDATA) [37] including 4,781 incident PD patients, 1,699 (36%) transfers to HD occurred, with a median time spent on PD of 358 days. The design of our study allows us to describe what happened after this transition. Notably, 506 of these 1,452 patients (35%) resumed PD as a third KRT modality (PD-HD-PD), probably after a temporary transfer, as the median time spent in HD was 9 days (Table 3). Even if data from the literature are reassuring regarding the outcome after a temporary HD transfer, we can wonder if this temporary transfer could be avoided in some patients with a residual renal function who maybe could stop dialysis for a period of less than 10 days [38].

We defined mixed dialysis as a short rotation between HD and PD. This definition and terminology has not been used previously in the literature, but we thought it might well describe a situation often encountered in clinical practice. Interestingly, we showed that even after such mixed dialysis, patients who returned to PD remained on this modality for a substantial period of time. The median time on PD was 339 days for the subgroup of patients treated by mixed dialysis and subsequently by PD; the time spent on PD was 433 days in the subgroup of patients treated by HD followed by mixed dialysis followed by PD. *Lanot et al.* showed that mechanical complications are the main cause of PD to HD transfer at the beginning of PD [39]. The causes of mixed dialysis were not available in our database. It can be hypothesized that patients who undergo mixed dialysis have PD-related mechanical complications. We showed that returning to PD is feasible even after a period of mixed dialysis. Nevertheless, the periods of alternation between HD and PD might affect patients’ quality of life, and it can be suggested that psychosocial support is mandatory to cope with this period [7,25,40,41].

In the literature, hybrid therapy is mainly used after a period of PD, to address ultrafiltration loss or dialysis inadequacy [19]. No international validated definition exists. Therefore, in our study, the definition we used was based on clinical practices, and also on the existing literature on that topic, mainly coming from Japan where this strategy is often used. The median time spent on hybrid therapy after initiation of PD was 386 days in our study; this strategy seemed to prolong the total time spent on home dialysis [42]. We showed that hybrid dialysis is underused in France, even though it has advantages. Further studies are needed to determine the obstacles to its development.

Our study has limitations: 12% of our patients were not matched according to the REIN-SNDS algorithm. This figure is consistent with other studies using this algorithm. Pre emptive transplants were excluded. Unfortunately, the causes of transfers were not available. Some specific data regarding medication and causes of hospitalization could be helpful to approach the causes of transfer. However, it is not possible with the extraction done for this study. The reconstruction of patient trajectories depends on the quality of coding of dialysis sessions in the SNDS. Some errors are possible, but only at the margin: like any other medico-administrative database, reimbursement of the dialysis sessions is linked to the coding. The calculation of the number of KRTs per patient included the mixed modality as a modality by itself. This may give the impression of overestimating the number of modalities per patient, compared with conventional registry data. This choice was made so that this very particular phase would be recognized in the patient’s pathway. Until now, this period has been invisible. Finally, the generalizability of our results to other countries can be questioned as each country has its own organization of KRTs, depending on the organization of the healthcare system and reimbursement arrangements [43]. For example, in France, the patient has no out-of-pocket expenses, via government health insurance. France is known to have a low PD prevalence, as only 6% of the prevalent patients are treated by PD [44], but a large coverage of all costs linked to dialysis, like nurse-assistance for PD for example [43]. We think that the care pathways depicted in the present study are influenced by different factors: organization of the healthcare system, complications and troubleshooting linked to PD itself and patients’ choice. The healthcare system of course varies a lot between countries, but we believe that trajectories depicted here are mainly driven by patients’ choice and inherent hazards linked to PD which may be similar in other countries. Therefore, we think that our results could be of interest for patients and nephrologists from other countries too.

Regarding future research, we think it would be interesting to replicate this work on hemodialysis patients (pathways between centers, home and satellite units). A qualitative study of the patient’s experience of the transfer would also be interesting. In this study we approach a new concept of mixed dialysis, which has not been defined previously. A qualitative study on the subject would be very interesting to have more feedback on the patients’ perception. Patients’ age and comorbidities probably influence their trajectory. A study on this subject would be desirable. Finally, it would be interesting to study the potential change in dialysis trajectories over time to see if the change in the healthcare system has had an impact on patients’ trajectories.

In conclusion, we showed that the care trajectories of PD patients are complex and variable. We reported that the detailed day by day dialysis regimen is much more complex than what we can see through data from conventional registries. HD and PD are complementary techniques that are frequently associated in the patient’s trajectory, either temporarily for acute complication or permanently in case of definitive transition. PD is a KRT that is the first foremost component of a multimodal pathway, as two thirds are likely to receive another KRT. Ongoing information about other KRTs seems necessary for PD patients.

## Supporting information

S1 Fig Examples of trajectories of patients, reconstructed from the SNDS database, showing how the creation of “mixed dialysis” and “hybrid dialysis” helps to summarize the trajectories of patients. (TIF)

S1 Table Characteristics of patients: overall population and PD-HD-PD patients. (XLSX)
