# Supplementary figures and images for: The diversity of peritoneal dialysis care trajectories: A study based on the REIN registry and SNDS database
Source: PLoS One. 2025 Aug 5;20(8):e0326745. doi: 10.1371/journal.pone.0326745 (PMC12324115; doi:10.1371/journal.pone.0326745)

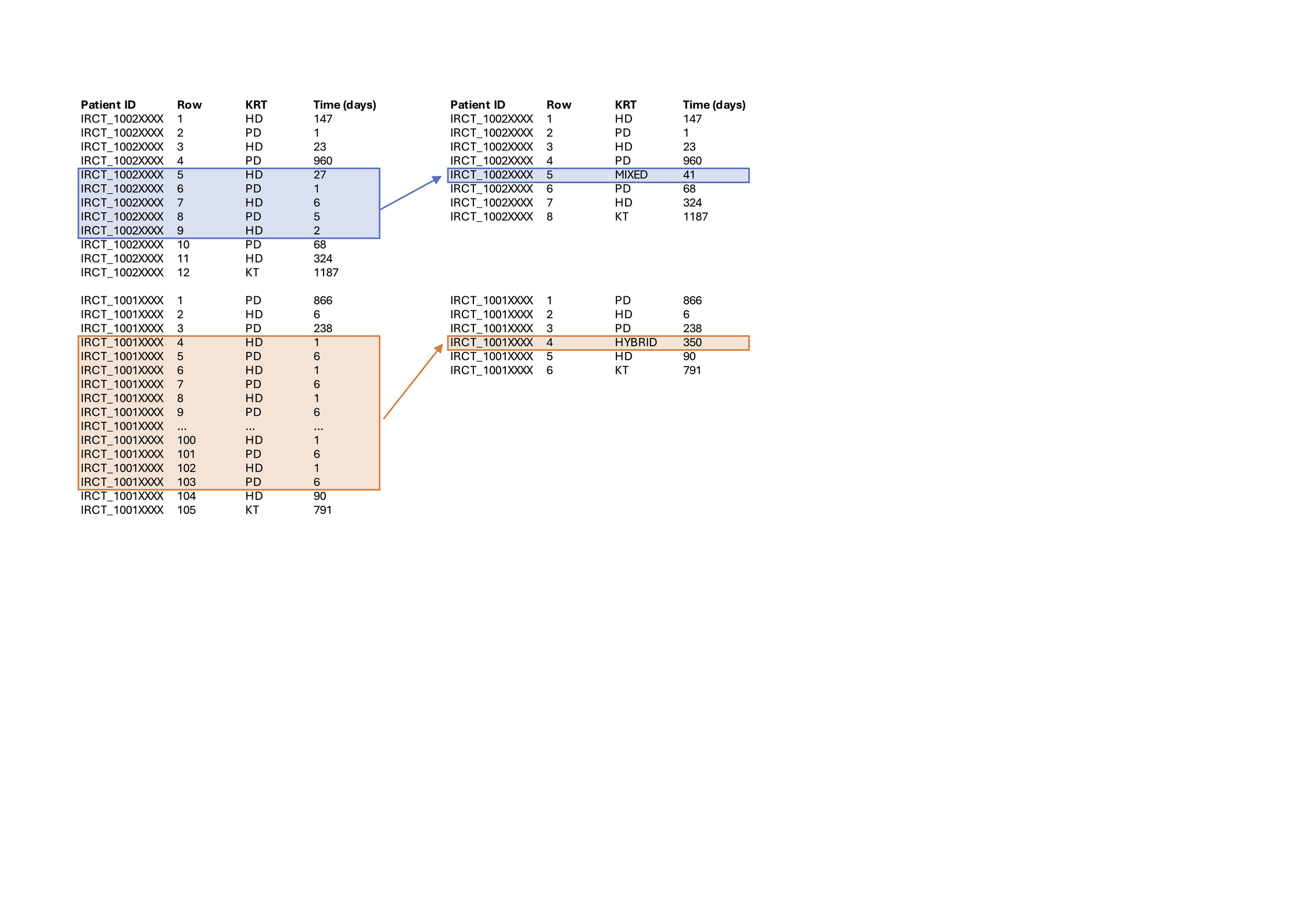

Supplement: S1 Fig — (TIF) [file pone.0326745.s001.tif]
